# Association between perceived stress and MAFLD partially mediated by smoking and drinking

**DOI:** 10.3389/fmed.2025.1569992

**Published:** 2025-07-29

**Authors:** Yan Gong, Shengshu Wang, Jianan Jiang, Qiang Zeng, Weimin Wang, Yansong Zheng, Wenping Lv

**Affiliations:** ^1^Department of Health Medicine, Second Medical Center and National Clinical Research Center for Geriatric Disease, Chinese PLA General Hospital, Beijing, China; ^2^Institute of Geriatrics, Beijing Key Laboratory of Ageing and Geriatrics, National Clinical Research Center for Geriatric Diseases, Second Medical Center of Chinese PLA General Hospital, Beijing, China; ^3^Faculty of Hepato-Pancreato-Biliary Surgery, First Medical Center, Chinese PLA General Hospital, Beijing, China

**Keywords:** metabolic-related fatty liver disease, perceived stress, physical inactivity, causal mediation analysis, smoking, drinking

## Abstract

**Background:**

Although the association between stress and NAFLD has been suggested, the effect of perceived stress on MAFLD has yet to be investigated. In this study, we explore the association between perceived stress and MAFLD.

**Methods:**

We performed a cross-sectional study including 36,847 subjects who underwent health check-ups from January 2011 to December 2021. MAFLD was defined as both fatty liver disease and metabolic dysfunction. The level of perceived stress was measured using the Chinese version of the 14-item Perceived Stress Scale (PSS-14). Logistic regression were performed to explore the association between perceived stress and MAFLD, and mediation analysis were used to examine smoking or drinking that may mediate the effects of perceived stress on MAFLD.

**Results:**

The prevalence of MAFLD was 37.10% (13,672/36,847). After controlling for sex, age, and BMI, the MAFLD incidence in subjects with a high level of perceived stress was significantly greater than that in subjects with a low level of perceived stress (40.4% vs. 34.9%) (*P* < 0.001). Perceived stress was positively associated with MAFLD [OR 1.076, 95% CI (1.005–1.153), *P* = 0.036]. MAFLD subjects with high perceived stress level exhibited higher rates of smoking, drinking and physical inactivity compared with non-MAFLD subjects. The mediation analysis revealed that the association between perceived stress and MAFLD was partially mediated by smoking and drinking, with a synergistic effect observed in individuals engaging in both behaviors.

**Conclusions:**

This study provided evidence for the potential association between perceived stress and MAFLD and the mediation analysis suggested the association of perceived stress and MAFLD was partially mediated by smoking and drinking. Public health strategies should target both smoking and drinking especially in high-stress populations, given their compounded risk for MAFLD.

## 1 Introduction

Non-alcoholic fatty liver disease (NAFLD) is one of the most common chronic liver diseases. It is estimated that the overall prevalence of NAFLD worldwide is as high as 32.4% (1). Recently, NAFLD was replaced with the new concept of metabolically related fatty liver disease (MAFLD) based on evidence of hepatic steatosis frequently accompanied by the presence of overweight/obesity, diabetes, or metabolic disorders (2, 3). MAFLD is associated with adverse outcomes of intrahepatic and extrahepatic diseases (such as cardiovascular disease and diabetes), making it an important global health and economic burden (4). Therefore, identifying potential modifiable risk factors and individuals at high risk of MAFLD are necessary.

Stress is considered a major public health challenge in modern society, and it increases the risk of obesity, metabolic syndrome, and various other chronic diseases (5, 6). The concept of stress can be roughly divided into environmental stress, biological stress and psychological stress. Studies have shown that psychological stress is related to lifestyles such as eating behavior, smoking and physical activity (7). There is mounting evidence of a possible association between perceived stress and NAFLD. Several studies have reported that a higher level of perceived stress was independently associated with a greater incidence of NAFLD, which remains significant after controlling for risk factors such as smoking, physical inactivity and alcohol consumption (8). Other studies have shown that perceived stress is not the direct cause of NAFLD, and stressful events lead people to choose unhealthy food and aggravate metabolic diseases through poor living habits such as overeating, excessive drinking and smoking. The interaction between morphopathological obesity, type 2 diabetes, metabolic syndrome and other metabolic diseases promotes NAFLD (9–11). Currently, there is no consensus on the interrelation between perceived stress and NAFLD. In addition, existing reports on perceived stress have used the traditional NAFLD standard (12), and studies on the association between perceived stress and MAFLD have been limited.

Therefore, the aims of the present study were to explore the clinical characteristics of MAFLD and the association between perceived stress and MAFLD in Chinese adults. And to further analyze whether perceived stress participated in MAFLD through the joint mediating effects of physical inactivity, smoking and alcohol consumption.

## 2 Methods

### 2.1 Study population

This retrospective study included 53,106 healthy individuals (36,198 males and 16,908 females) who underwent physical examination and completed a questionnaire at the Health Management Institute, PLA General Hospital, from January 2011 to December 2021. The questionnaires were sent before the participants' health check-up and included information on lifestyle, perceived stress and medical history. First, we excluded 10,516 participants who had repeated physical examinations and 3,927 participants who had incomplete questionnaire information. In addition, 1,816 patients with missing platelet counts (*n* = 1,420), missing fasting blood glucose information (*n* = 46), incomplete liver function indices (*n* = 187) or missing drinking information (*n* = 163) were excluded. A total of 36,847 consecutive subjects were enrolled in the final study. The flow chart of the study design is illustrated in Supplementary Figure S1. The study protocol was approved by the Ethics Committee of the General Hospital of the People's Liberation Army of China (No. S2022-720). Written informed consent was obtained from each enrolled individual.

### 2.2. Clinical and biochemical evaluation

The collected data included demographic information, daily lifestyle information, laboratory findings and medical history. Lifestyle information was obtained through questionnaires. Waist circumference (WC) was measured with a tape measure around the body at the midpoint between the lower costal margin of the waist and the anterior superior iliac crest. Weight and height were measured using a body composition analyzer, and BMI was calculated as weight (kg)/height (m^2^). Overweight was defined as a body mass index (BMI) between 24 and 28 kg/m^2^, and obesity was defined as a BMI ≥ 28 kg/m^2^ according to the 2016 “Expert consensus on mediconutritional treatment of overweight and obesity in China” (13). Individuals who smoked were classified as current smokers (smoking ≥10 cigarettes per day, continuous for at least 1 year) or non-current smokers (continuous cessation of smoking for more than 1 year) (14). Alcohol consumption was categorized as moderate drinking (drinking 1–19 g per day) or excessive drinking (drinking 20 g or more per day on average) (15). The level of education was divided into (1) primary school and below, (2) secondary education (junior high school, senior high school, technical secondary school), and (3) higher education (junior college, university undergraduate, postgraduate). We divided physical activity into (1) insufficient physical activity: participants who did not participate in any level of physical activity per week or <30 min of average physical activity per session; and (2) physical activity: 1–2, 2–3 or more times per week or 30–60 min or more of average physical activity per session. Blood pressure was measured twice by an electronic sphygmomanometer and averaged. Hypertension was defined as a blood pressure ≥140/90 mmHg or history of antihypertensive drug use; diabetes was defined as a fasting blood glucose level ≥7 mmol/L, an HbA1c level ≥6.5%, or a history of hypoglycemic drug use (16, 17). Hyperlipidemia was defined as a serum cholesterol concentration ≥5.72 mmol/dL, triglyceride (TG) concentration ≥1.70 mmol/dl, or high-density lipoprotein cholesterol (HDL-C) concentration <0.91 mmol/L (18). Metabolic syndrome was defined as having at least three of the following: waist circumference >102 cm for men or >88 cm for women, fasting blood glucose ≥6.10 mmol/L, blood pressure ≥130/85 mmHg, elevated TG ≥1.70 mmol/L, and HDL-C (< 1.04 mmol/dl in men or 1.29 mmol/dl in women) (19).

Venous blood (5 ml) was collected after the patients had fasted for at least 12 h for quantification of biochemical parameters in accordance with the quality control and detection standards of the Clinical Laboratory of the General Hospital of the People's Liberation Army. Serum triglycerides, fasting blood glucose, fasting insulin, high-density lipoprotein cholesterol, aspartate aminotransferase (AST), alanine aminotransferase (ALT), total cholesterol (TC), low-density lipoprotein cholesterol (LDL-C), and high-density lipoprotein cholesterol (HDL-C) were determined using commercially available reagents (Roche Diagnostics).

### 2.3 Diagnosis of MAFLD

Abdominal ultrasounds were performed by 12 experienced radiologists who were unaware of the study aims using ultrasound scanners (Acuson Sequoia 512; Siemens, Mountain View, CA, USA). The diagnosis of fatty liver disease relies mainly on increased echogenicity in the liver and kidney, contrast between the liver and kidney parenchyma, attenuation of the deep ultrasonic beam, blurring of blood vessels and hepatic venous stenosis (20). Each subject was further staged as mild fatty liver, moderate fatty liver, or severe fatty liver. Mild steatosis was seen as a slight increase in liver echogenicity. In moderate steatosis, visualization of intrahepatic vessels and the diaphragm was slightly impaired, and increased liver echogenicity was present. Severe steatosis was recognized as a marked increase in hepatic echogenicity, poor penetration of the posterior segment of the right lobe of the liver, and poor or no visualization of the hepatic vessels and diaphragm (21).

The diagnosis of MAFLD was based on the diagnostic criteria of the Asian population proposed by an international expert consensus, that is, the diagnosis of fatty liver (mild, moderate, or severe) by ultrasound and included overweight or obesity (as determined by BMI overweight as 24 to < 28 kg/m^2^, and obesity as ≥28 kg/m^2^) (22), type 2 diabetes or metabolic dysfunction (including increased waist circumference, abnormally elevated blood pressure, abnormally elevated triglycerides, abnormally elevated HDL cholesterol, prediabetes, and abnormally elevated HOMA-IR of at least 2 risk factors for metabolic abnormalities); meeting at least one of the three criteria was needed (2). The FIB-4 index was used to evaluate hepatic fibrosis in patients with MAFLD. An FIB-4 index score ≥1.3 was an indicator of hepatic fibrosis (23).

### 2.4 Perceived stress assessment

The Chinese version of the 14-item PSS (PSS-14) was used to measure the perceived level of stress (24). The scale has been proven to have good reliability and validity, and its coefficient is 0.78. The correlation coefficient between the total score of the scale and each item ranged from 0.37 to 0.53, and the surface homogeneity and internal consistency was high (25). There are 14 items in the scale and each item is rated on a five-point scale ranging from 0 = “never” to 4 = “very often”. The scale can cluster into two subscales: negative subscale (items 1, 2, 3, 8, 11, 12, and 14) and positive subscale (items 4, 5, 6, 7, 9, 10, and 13). The total score of PSS is obtained by reversing the scores on the positive items and then summing across all the items, with a higher score indicating higher perceived stress. The total scores for PSS-14 range from 0 to 56, and scores higher than 28 were set as high perceived stress.

### 2.5 Statistical analysis

All normally distributed variables are presented as the mean and standard deviation (SD), and categorical variables are presented as the frequency and percentage. All non-normally distributed variables are presented as medians and interquartile ranges (IQRs). Student's *t* test and ANOVA were used to analyze continuous variables, and the differences between nominal variables were compared with the chi-square test. Logistic regression analysis was then performed to analyze the association between perceived stress and MAFLD incidence after adjusting for potential confounders. The causal mediation analysis was performed using EmpowerStats, based on a counterfactual framework with the following assumptions: (1) no unmeasured confounding between the exposure (perceived stress) and the outcome (MAFLD), (2) no unmeasured confounding between mediators (smoking/drinking) and the outcome, and (3) no exposure-mediator interaction. We used a non-parametric bootstrapping approach (5,000 resamples) to estimate confidence intervals, which does not assume linearity in the relationships. Sensitivity analyses were conducted to assess potential unmeasured confounding by varying mediator-outcome confounding scenarios, showing that our results remained robust unless an unmeasured confounder had an implausibly strong effect (OR > 2.5). All the statistical analyses were performed using SPSS 22.0 software (SPSS, Inc., Chicago, Illinois, USA), and *P* values < 0.05 were considered to indicate statistical significance.

## 3 Results

### 3.1. Baseline characteristics of the study population

A total of 36,847 subjects, including 13,672 subjects with MAFLD and 23,175 controls, were included in the final analysis. The mean age was 47.25 years (SD, 8.24 years) and the male proportion was 66.4%. The prevalence rate of MAFLD was 37.10% (13,672/36,847). The baseline characteristics of the participants according to MAFLD status are shown in Table 1. MAFLD was more frequently observed in males, individuals with higher education levels, smokers and drinkers. The incidence of hypertension was significantly greater in the MAFLD group than in the non-MAFLD group, and more individuals had FIB-4 scores ≥1.3. In addition, most anthropometric and laboratory indices, including BMI, waist circumference, aspartate transaminase (AST), alanine transaminase (ALT), fasting blood glucose, total cholesterol, triglyceride, high-density lipoprotein cholesterol (HDL-C), and apolipoprotein levels, were associated with poorer metabolic characteristics in the MAFLD group (*P* < 0.001).

**Table 1:** Demographic and clinicopathological characteristics of the participants.

**Feature**	**MAFLD**	**Non-MAFLD**	***P*-value**
Number (%, n/n)	37.1% (13,672/36,847)	62.9% (23,175/36,847)	N/A
Age (years)	48 (43–53)	47 (42–52)	<0.001^*^
Sex (female/male)	16.0%/84.0% (2,194/11,478)	43.9%/56.1% (10,174/13,001)	<0.001^*^
γ.GT (U/L)	41 (27–68)	22 (14–38)	<0.001^*^
ALT (U/L)	27.1 (19.5–38.9)	17.3 (12.6–24.7)	<0.001^*^
AST (U/L)	20.4 (16.9–25.7)	17.6 (14.9–21.2)	<0.001^*^
TB (μmol/L)	10.9 (8.3–14.3)	10.1 (7.5–13.6)	<0.001^*^
TP (g/L)	71.2 (67.8–74.6)	70.8 (66.9–74.5)	0.762
ALB (g/L)	46.2 (44.0–48.2)	45.6 (43.2–47.8)	0.001^*^
DB (μmol/L)	3.6 (2.9–4.6)	3.5 (2.7–4.4)	<0.001^*^
ALP (U/L)	66 (56–79)	61 (51–74)	0.007^*^
C-P (ng/ml)	3 (2.5–3.7)	2.1 (1.7–2.6)	<0.001^*^
INS (mU/L)	12.7 (9.3–17.6)	7.8 (5.6–10.8)	0.040^*^
FPG (mmol/L)	5.7 (5.2–6.4)	5.2 (4.9–5.6)	<0.001
LDL (mmol/L)	3.2 (2.6–3.8)	3.1 (2.5–3.6)	0.554
CHO (mmol/L)	4.9 (4.3–5.5)	4.7 (4.1–5.3)	<0.001^*^
TG (mmol/L)	2.0 (1.4–2.9)	1.3 (0.9–1.8)	<0.001^*^
Lp(a) (mg/L)	13.5 (6.1–30.7)	17.0 (7.9–39.0)	<0.001^*^
apo.A1 (g/L)	1.3 (1.1–1.4)	1.4 (1.2–1.5)	<0.001^*^
apo.B. (g/L)	1.0 (0.9–1.1)	0.9 (0.8–1.1)	<0.001^*^
apo.E (g/L)	5.0 (4.1–6.3)	4.5 (3.8–5.4)	0.869
HDL (mmol/L)	1.1 (0.9–1.2)	1.3 (1.1–1.5)	0.003^*^
BMI (kg/m^2^)	27.2 (25.5–29.2)	23.7 (21.9–25.1)	<0.001^*^
BFR (%)	28.0 (24.9–31.7)	26.1 (22.4–30.0)	<0.001^*^
Waist (large/normal)	95 (90–100)	84 (77–91)	0.016^*^
Educational background (low/moderate/high)	18%/16.1%/65.9% (2,215/1,983/8,096)	20.4%/15.7%/63.9% (4,246/3,265/13,303)	<0.001^*^
Smoking (No/Yes)	50.1%/49.9% (6,854/6,818)	67.3%/32.7% (15,605/7,570)	<0.001^*^
Drinking (No/Yes)	25.2%/74.8%(3,442/10,230)	42.0%/58.0%(9,727/13,448)	<0.001^*^
Exercise frequency (low/moderate/high)	48.2%/16.4%/35.4 (4,201/1,434/3,086)	48.6%/17.0%/34.4% (6,651/2,324/4,697)	<0.001^*^
Exercise duration (low/moderate/high)	21.7%/15.7%/43.1%/19.5% (1,393/1,010/2,773/1,255)	21.7%/15.3%/43.6%/19.4% (2,175/1,532/4,372/1,943)	<0.001^*^
Pressure (low/high)	57.7%/42.3% (7,896/5,776)	63.2%/36.7% (14,667/8,508)	<0.001^*^
Hypertension (No/Yes)	60.0%/40.0% (7,611/5,983)	32.6%/67.4% (7,501/15,533)	<0.001^*^
FIB-4	0.9 (0.7–1.1)	0.9 (0.7–1.1)	0.005^*^

MAFLD, metabolic-associated fatty liver disease; r-GT, r -glutamyltransferase; ALT, alanine aminotransferase; AST, aspartate aminotransferase; TB, total bilirubin; ALB, albumin; DB, direct bilirubin; ALP, alkaline phosphatase; C-P, C-peptide; INS, insulin assay; FPG, fasting plasma glucose; LDL, low-density lipoprotein cholesterol; CHO, total cholesterol; TG, triglyceride; Lpa, Lipoprotein a; apo, apolipoprotein; HDL, high-density lipoprotein cholesterol; BMI, body mass index; BFR, body fat rate. ^*^P < 0.05.

### 3.2. Comparisons of MAFLD incidence and perceived stress stratified by sex, age, and BMI

**Figure 1:**
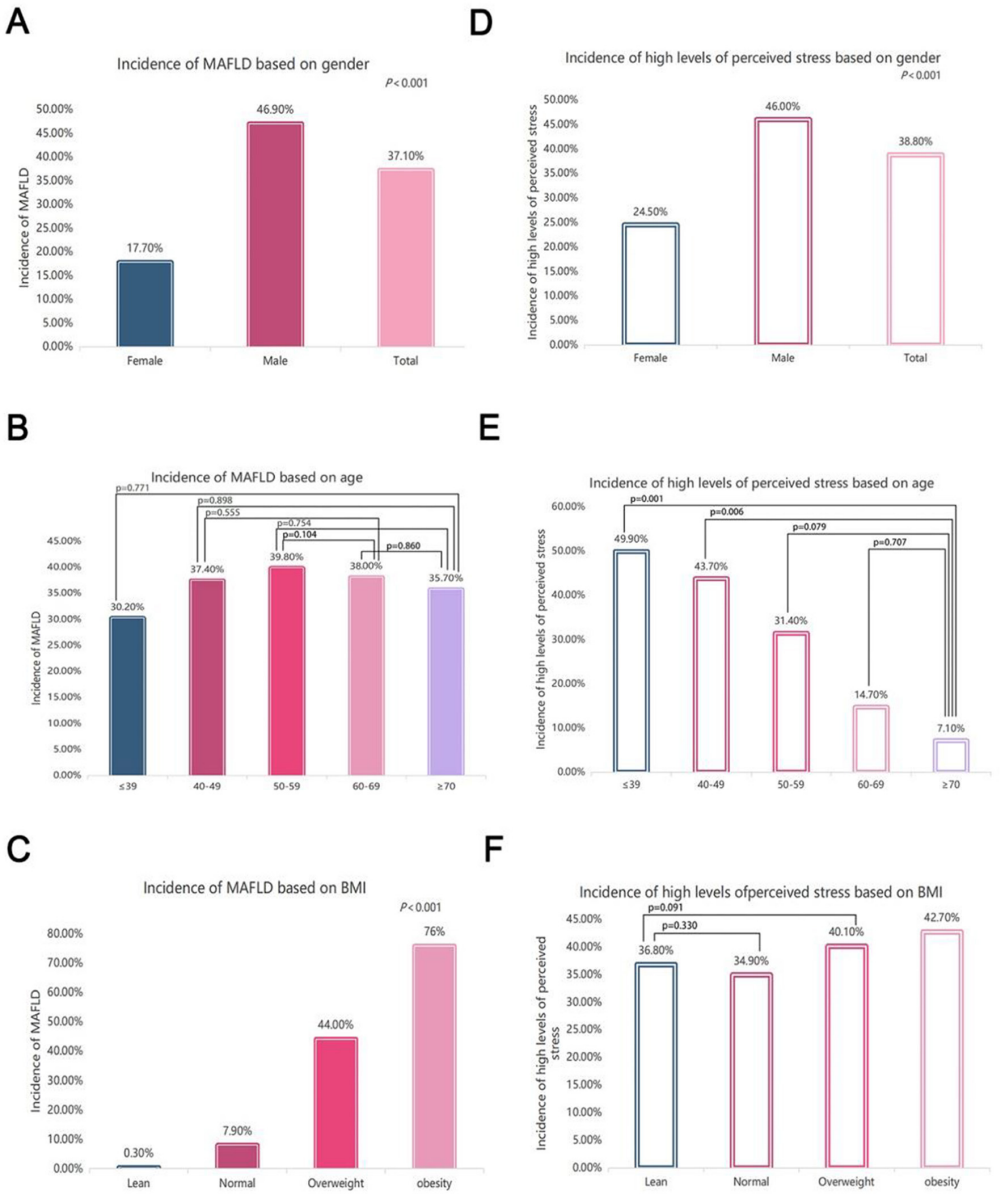
Comparisons of MAFLD incidence and perceived stress levels stratified by sex, age, and BMI. **(A)** Overall incidence of MAFLD in the subjects and comparison of the MAFLD incidence between males and females (the difference between each group was statistically significant, *P* < 0.001); **(B)** comparison of the MAFLD incidence among the subjects stratified by age after controlling for sex and BMI (except for the *P* value marked in the figure, the comparison difference between the other two groups was statistically significant, *P* < 0.05); **(C)** comparison of the MAFLD incidence among the subjects stratified by BMI after controlling for sex and age (the difference between each group was statistically significant, *P* < 0.001); **(D)** comparison of the incidence of high perceived stress levels between males and females (the difference between each group was statistically significant. *P* < 0.001); **(E)** comparison of the incidence of high perceived stress levels among the subjects stratified by age after controlling for sex and BMI (except for the *P* value marked in the figure, the difference between the other two groups was statistically significant, *P* < 0.05); **(F)** comparison of the incidence of high perceived stress levels among the subjects stratified by BMI after controlling for sex and age (except for the *P* value marked in the figure, the difference between the other two groups was statistically significant, *P* < 0.05).

The comparisons of MAFLD incidence and perceived stress levels stratified by sex, age, and BMI are presented in Figure 1. The prevalence of MAFLD in all subjects was 37.10%, and the 46.90% prevalence in males was significantly greater than the 17.70% prevalence in females after adjusting for age and BMI (*P* < 0.001) (Figure 1A). There was no significant difference in the MAFLD incidence among subjects stratified by age (Figure 1B). The incidence of MAFLD markedly increased with increasing BMI (*P* < 0.001 for trend) (Figure 1C). Additionally, the incidence of high perceived stress levels was significantly greater in males than in females (*P* < 0.001) (Figure 1D). The incidence of high perceived stress levels decreased with increasing age. The incidence was greater in subjects < 50 years than in subjects ≥50 years (Figure 1E). The incidence of high perceived stress levels was greater in obese subjects than in subjects with a normal BMI (Figure 1F).

### 3.3 Comparisons of MAFLD prevalence

**Figure 2:**
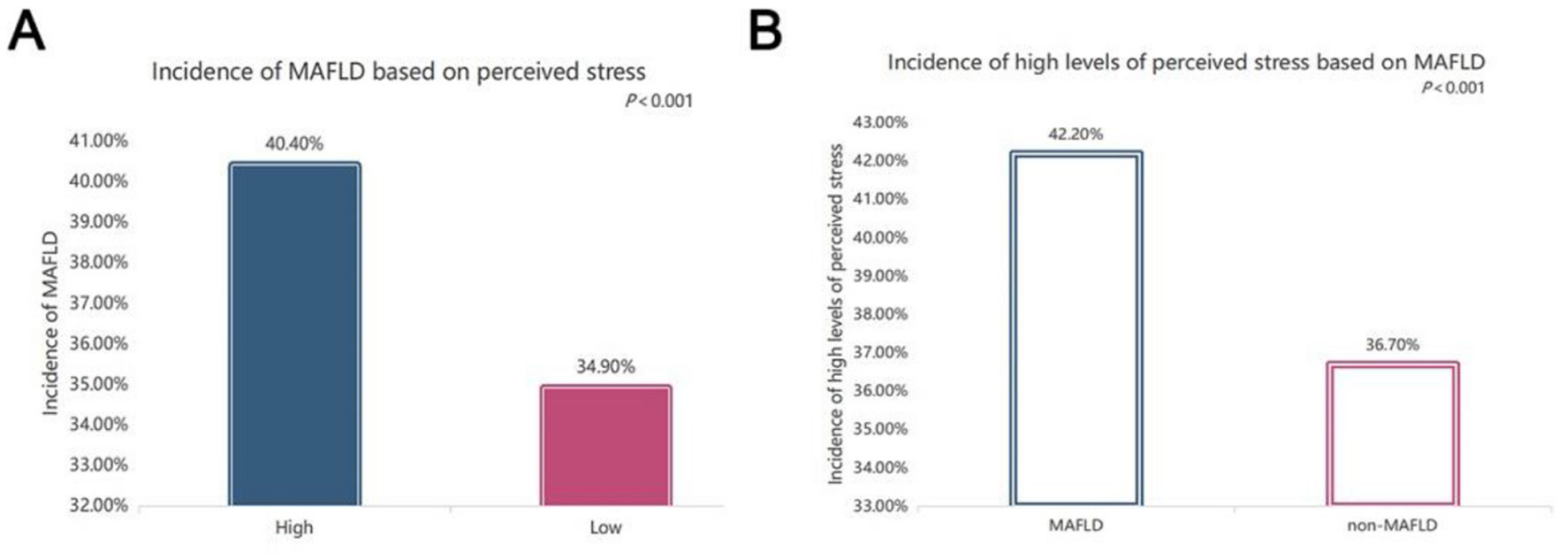
Comparisons of the prevalence of metabolic dysfunction-associated fatty liver disease (MAFLD). **(A)** Comparisons of MAFLD incidence based on different perceived stress levels after controlling for sex, age, and BMI (*P* < 0.001); **(B)** comparisons of the incidence of high levels of perceived stress between subjects with and without MAFLD (*P* < 0.001).

Figure 2 displays the comparisons of MAFLD incidence among the different perceived stress levels and the difference of perceived stress levels between subjects with and without MAFLD. After adjustment for sex, age and BMI, the incidence of MAFLD in subjects under high-level perceived stress was 40.4%, which was significantly greater than that in subjects under low-level perceived stress (34.9%) (*P* < 0.001) (Figure 2A). Additionally, the incidence of high perceived stress was significantly greater in MAFLD subjects than in non-MAFLD controls (42.3% vs. 36.7%, *P* < 0.001) (Figure 2B).

### 3.4 Comparisons of liver enzymes

**Figure 3:**
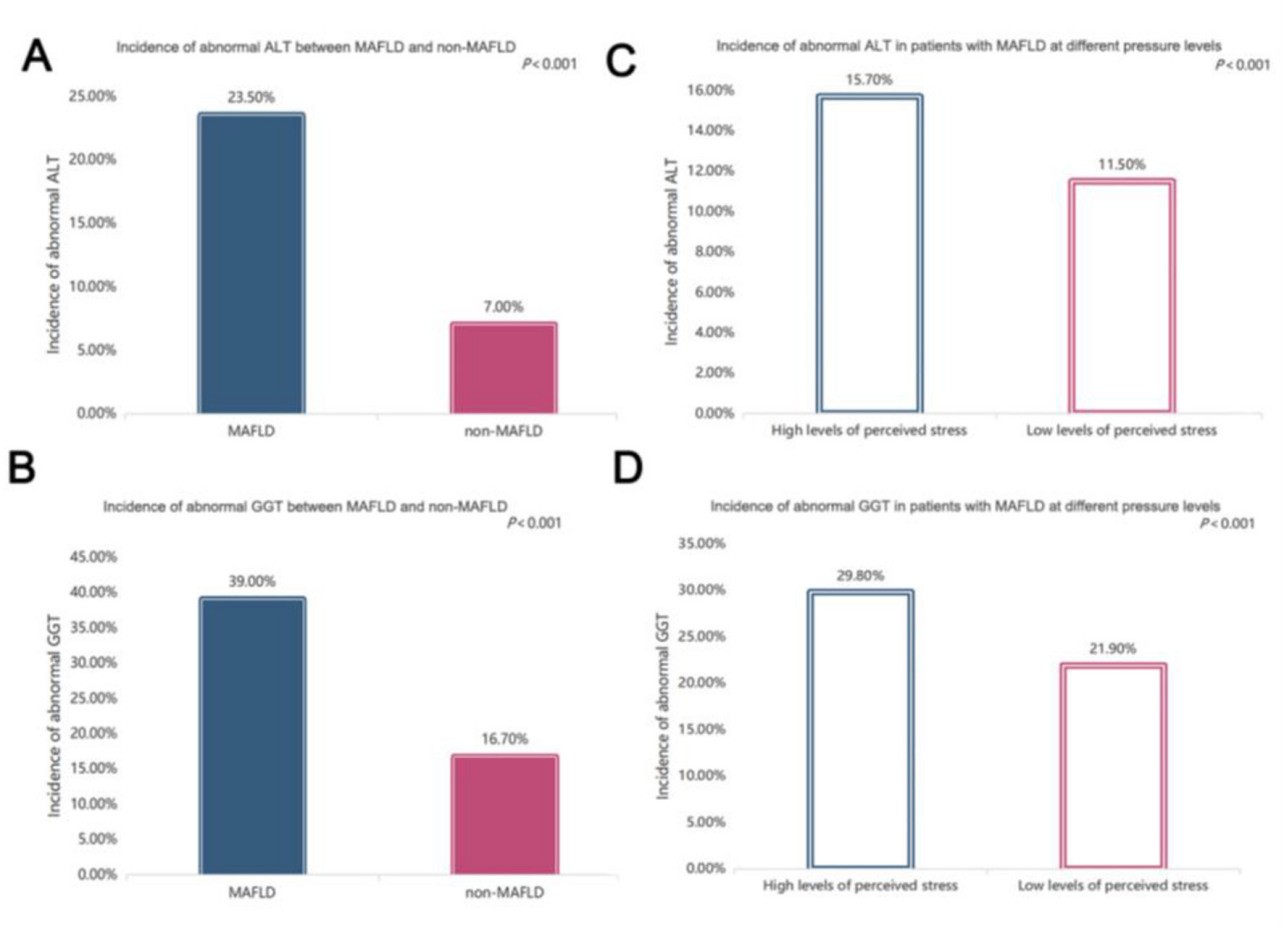
Comparisons of liver enzymes. **(A)** Comparisons of alanine aminotransferase (ALT) levels between subjects with and without metabolic dysfunction-associated fatty liver disease (MAFLD) after controlling for age, sex, and BMI (*P* < 0.001); **(B)** comparisons of ALT levels across the different levels of perceived stress after controlling for age, sex, and BMI (*P* < 0.001); **(C)** comparisons of γ-glutamyltransferase (γ-GT) levels between subjects with and without MAFLD after controlling for age, sex, and BMI (*P* < 0.001); **(D)** comparisons of γ-GT levels across the different levels of perceived stress after controlling for age, sex, and BMI (*P* < 0.001).

Figure 3 compares liver enzymes, including ALT and γ-GT, among the different perceived stress levels. As shown in Figures 3A, B, both the serum ALT and γ-GT levels in the MAFLD patients were significantly greater than those in the non-MAFLD controls (all *P* < 0.001). Furthermore, both the serum ALT (*P* = 0.007 for trend) and γ-GT levels (*P* < 0.001) were clearly lower in subjects with low levels of perceived stress than in those with high levels of perceived stress (Figures 3C, D).

### 3.5 Comparisons of exercise, smoking and alcohol consumption

**Figure 4:**
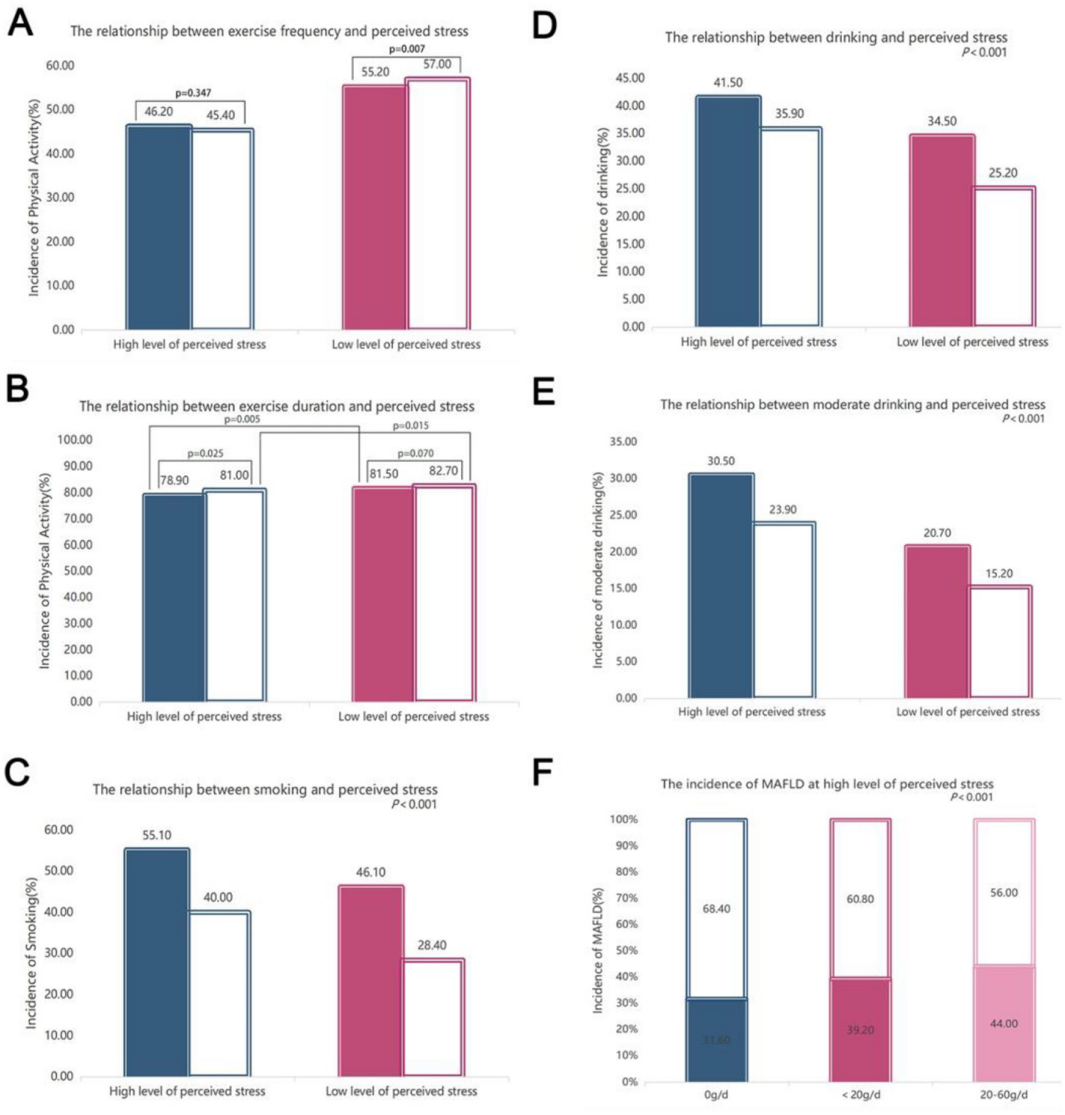
Comparisons of exercise, smoking and drinking status. **(A)** Comparison of the frequency of exercise associated with different levels of perceived stress with MAFLD after controlling for age, sex, and BMI (*P* < 0.001); **(B)** comparison of the duration of exercise associated with different levels of perceived stress with and without MAFLD after controlling for age, sex, and BMI (*P* < 0.001); **(C)** comparison of smoking under different levels of perceived stress with and without MAFLD after controlling for age, sex, and BMI (*P* < 0.001); **(D)** comparison of drinking under different levels of perceived stress with and without MAFLD after controlling for age, sex, and BMI (*P* < 0.001); **(E)** comparison of moderate drinking under different levels of perceived stress with and without MAFLD after controlling for age, sex, and BMI (*P* < 0.001); **(F)** comparison of the incidence of MAFLD in subjects with high levels of perceived stress under different alcohol consumption conditions (*P* < 0.001). For high and low levels of perceived stress, the left group is MAFLD, and the right group is non-MAFLD. For the comparisons of the other two groups, *P* < 0.001 was used, except for the *P* value indicated in the figure.

We compared the frequency and duration of exercise between MAFLD individuals with low and high perceived stress. 46.2% of MAFLD individuals with a high level of perceived stress exercised, compared with the MAFLD individuals with a low level of perceived stress (55.2%, *P* = 0.007), had lower exercise frequency every week (Figure 4A). 78.9% of MAFLD individuals with a high level of perceived stress exercised, compared with the proportion of MAFLD individuals with a low level of perceived stress (81.5%, *P* = 0.005) and that of non-MAFLD individuals with a high level of perceived stress (81%, *P* = 0.025), had less exercise duration (Figure 4B).

Overall, 55.1% of MAFLD individuals with a high level of perceived stress smoked, compared with MAFLD individuals with a low level of perceived stress (46.1%, *P* < 0.01), the proportion was increased. Furthermore, the number of smokers who had MAFLD and a high level of perceived stress was significantly greater than the number of non-smokers with MAFLD and a high level of perceived stress (40%, *P* < 0.01) (Figure 4C).

A total of 41.5% and 30.5% of MAFLD individuals with a high level of perceived stress have little and moderate drinking, respectively, which were significantly greater than those of MAFLD subjects with low levels of stress (including 34.5% of drinkers and 20.7% of moderate drinkers) (Figure 4D). More MAFLD individuals with high levels of stress drink compared with the non-MAFLD patients with high levels of stress (41.5% vs. 35.9%, *P* < 0.001). Additionally, compared with non-MAFLD controls, MAFLD patients with high levels of stress also had an increased risk of moderate drinking (30.5% vs. 23.9%, *P* < 0.001) (Figure 4E). Among those subjects with a high level of perceived stress, non-drinkers, moderate drinkers and excessive drinkers accounted for 31.6%, 39.2% and 44%, respectively, and the incidence of MAFLD increased with increasing alcohol consumption (Figure 4F).

### 3.6 Association of perceived stress with MAFLD

Next, we analyzed the potential association between MAFLD and perceived stress. Univariate analysis revealed that the presence of high levels of perceived stress was significantly associated with the incidence of MAFLD, and compared with individuals with low levels of stress, those with high levels of perceived stress had a 26.1% increased risk of MAFLD [odds ratio (OR) 1.261, 95% confidence interval (CI), 1.208–1.317; *P* < 0.001]. After adjusting for age, sex, smoking status, alcohol consumption, physical inactivity, blood pressure, waist circumference, gallbladder polyps, and cholesterol levels according to multivariate analyses, high levels of perceived stress were still associated with MAFLD [OR 1.076, 95% CI, (1.005–1.153); *P* = 0.036]. Additionally, male [OR 2.693, 95% CI (2.461–2.947), *P* < 0.05], smoking [OR 1.092, 95% CI (1.017–1.174), *P* = 0.016], physical inactivity [OR 1.198, 95% CI (1.104–1.300), *P* < 0.05], hypertension [OR 1.624, 95% CI (1.522–1.732), *P* < 0.001], HDL-C level [OR 2.053, 95% CI (1.911–2.205), *P* < 0.05], waist circumference [OR 5.047, 95% CI (4.700–5.421), *P* < 0.05], gallbladder polyps [OR 1.192, 95% CI (1.079–1.317), *P* = 0.001], gallstones [OR 1.202, 95% CI (1.075–1.345), *P* = 0.001], and cholesterol [OR 1.471, 95% CI (1.358–1.594), *P* < 0.05] were also significantly associated with an increased risk of MAFLD (Table 2). Smoking was associated with a 9.2% increased risk [OR 1.092, 95% CI (1.017–1.174)], while drinking was associated with a higher risk [OR 2.150, 95% CI (2.052–2.252)]. We performed additional analysis to evaluate the combined effect of smoking and drinking. The results showed that individuals who both smoke and drink have a synergistic risk increase (OR = 1.341, 95% CI: 1.243–1.448), which is higher than the sum of their individual risks (Supplementary Table 1).

**Table 2:** Univariate and multivariate analysis of perceived stress and MAFLD.

**Factors**	**Univariate analysis OR (95% CI)**	***P*-value**	**Multivariate analysis OR (95% CI)**	***P*-value**
Age	1.016 (1.014–1.019)	<0.001^*^		
Gender (Female)	4.094 (3.884–4.315)	<0.001^*^	2.693 (2.461–2.947)	<0.001^*^
Pressure	1.261 (1.208–1.317)	<0.001^*^	1.076 (1.005–1.053)	0.036^*^
Smoking	2.051 (1.964–2.141)	<0.001^*^	1.092 (1.017–1.174)	0.016^*^
Drinking	2.150 (2.052–2.252)	<0.001^*^		
Physicial inactivity	1.118 (1.041–1.200)	0.002^*^	1.198 (1.104–1.300)	<0.001^*^
Waist	6.377 (6.056–6.716)	<0.001^*^	5.047 (4.700–5.421)	<0.001^*^
Hypertension	2.634 (2.522–2.752)	<0.001^*^	1.624 (1.522–1.732)	<0.001^*^
HDL	3.133 (2.988–3.286)	<0.001^*^	2.053(1.911–2.205)	<0.001^*^
TG	1.143 (1.010–1.295)	0.035^*^		
ALP	1.220 (0.932–1.596)	0.147		
CHO	1.471 (1.395–1.552)	<0.001^*^	1.471 (1.358–1.594)	<0.001^*^
Gallbladder polyps	1.037 (0.969–1.109)	0.293	1.192 (1.079–1.317)	0.001^*^
Gallstone	1.456 (1.351–1.570)	<0.001^*^	1.202 (1.075–1.345)	0.001^*^

HDL, high-density lipoprotein cholesterol; TG, triglyceride; ALP, alkaline phosphatase; CHO, total cholesterol; ^*^P < 0.05.

To assess the potential impact of unmeasured confounding (e.g., diet, sleep disorders, or depression), we calculated *E*-values for the association between perceived stress and MAFLD. The *E*-value for the observed OR (1.076) was 1.21, indicating that an unmeasured confounder would need to be associated with both perceived stress and MAFLD by an OR of at least 1.21 to fully explain away the observed effect. For the lower CI bound (1.005), the *E*-value was 1.01, suggesting weaker unmeasured confounding would suffice to nullify the association. These *E*-values imply that only modest unmeasured confounding could attenuate our results, though stronger confounders (e.g., depression, which often correlates with stress at OR > 2.0) might partially account for the association.

### 3.7 Mediation effect of smoking, drinking and exercising on the association between perceived stress and MAFLD

Table 3 shows the causal mediation analysis results of exercise, smoking, and drinking on the relationship between perceived stress and MAFLD. In this population, causal mediation analysis revealed that smoking mediated 85.4% and drinking mediated 94.2% of the total effect of perceived stress on MAFLD.

**Table 3:** Causal mediation analysis of associations of perceived stress and MAFLD mediated by smoking, drinking and exercising.

**Factors**	**Estimate**	**95% CI**	***P*-value**
**Smoking**			
Direct effect (average)	0.041	0.031–0.052	<0.0001
Proportion mediated (average)	0.239	0.194–0.305	<0.0001
**Drinking**			
Direct effect (average)	0.029	0.019–0.039	<0.0001
Proportion mediated (average)	0.473	0.391–0.584	<0.0001
**Exercising**			
Direct effect (average)	0.054	0.0434–0.064	<0.0001
Proportion mediated (average)	0.015	−0.005–0.038	0.1300

## 4 Discussion

In this retrospective study, we found that the incidence of MAFLD increased with elevated perceived stress, and multivariate analysis suggested that high levels of perceived stress were positively correlated with MAFLD, partially mediated by smoking and excessive alcohol consumption. To our knowledge, this is the first report on the relationship between perceived stress and MAFLD in a check-ups population from China.

The prevalence of MAFLD in our study was 37.10%, and its prevalence was significantly associated with male sex. Like in some of our findings, Huang et al. (26) showed that significant sex differences influenced the incidence of MAFLD in young and middle-aged adults. We found that the incidence of MAFLD was positively correlated with BMI and was greater in males than in females. A recent study conducted by Sarkar et al. (27) showed that reduced testosterone levels in obese men were independently associated with fatty liver disease and may partially explain this phenomenon; however, further analysis is needed to understand these mechanisms accurately.

In our study of China (from the Han population district), we found that more than 1/3 of the asymptomatic population had high perceived stress, and the proportion of the population with a high level of perceived stress was greater, reaching 42.2%, among the MAFLD population. An explicit correlation was confirmed between perceived stress and MAFLD; additionally, men were more likely to suffer from MAFLD during stress, accounting for 92.0% of the patients. Multivariate regression analysis revealed that the risk of MAFLD in individuals with high levels of perceived stress was 7.6% greater than that in individuals without obvious perceived stress. The identification of perceived stress positively associated with MAFLD was consistent with the findings of previous studies on NAFLD. For example, the study from Han et al. (28) on the relationships between NAFLD and eating habits, stress and health-related quality of life (HRQoL) and reported that the risk of NAFLD increased by 1.316-fold with increasing stress. Another large cohort study designed to investigate the relationship between perceived stress and the incidence of NAFLD in apparently healthy men and women showed that higher perceived stress was independently associated with an increased incidence of NAFLD, and the association was stronger in men and obese individuals than in women and normal-weight individuals. The association remained significant after researchers adjusted for a variety of metabolic, behavioral, and socioeconomic factors (8).

Serum ALT and γ-GT have been used as sensitive biomarkers for assessing hepatocyte damage and are generally elevated in patients with MAFLD. In this study, both the serum ALT and γ-GT levels increased with increasing perceived stress. A previous study using foot shock to model chronic stress in mice revealed that chronic stress can lead to increased triglyceride and total cholesterol levels in mice, accompanied by microvesicular steatosis, lobular inflammation, and balloon-like degeneration (29). A clinical study of 567 patients with biopsy-confirmed NAFLD revealed a dose-dependent relationship between the severity of depressive mood and the degree of hepatocyte swelling (30). Our results further confirm the findings of previous research.

Our study confirmed that perceived stress contributes to unhealthy behaviors (including physical inactivity, smoking, and alcohol consumption) and that they together play a role in MAFLD. First, we found that increased perceived stress led to decreased exercise, and MAFLD individuals with high levels of perceived stress were more likely to lack exercise. These findings are similar to a previous report that also indicated that individuals with NAFLD and depression were more likely to be physically inactive than individuals without NAFLD and depression (31). This study also demonstrated that increased perceived stress was associated with increased smoking, and MAFLD patients with high levels of stress smoked more cigarettes. This may be because smokers are most likely to smoke instead of eating to cope with stress. Cigarettes contain a variety of carcinogens, such as polycyclic aromatic hydrocarbons, which can be directly absorbed by the liver mucosa or the circulation system, thereby increasing the risk of MAFLD (32, 33). Moreover, a high level of stress leads to excessive drinking, which is more obvious in MAFLD patients with a high level of stress. The proportions of individuals with excessive and moderate alcohol consumption increased by 12.8% and 5.2%, respectively, compared with individuals who did not drink alcohol. The reason may be that people with a high level of stress escape to cope with stress and may use drinking as a coping strategy (34–36). Our findings indicate that drinking contributes more significantly to MAFLD risk than smoking. Furthermore, the combined effect of smoking and drinking is multiplicative, with individuals engaging in both behaviors facing increased risk (OR = 1.341) compared to those who only drink or smoke. This suggests that the interaction between smoking and drinking exacerbates MAFLD risk beyond simple additive effects, possibly due to compounded metabolic and inflammatory pathways. Finally, causal mediation analysis indicated that the association between pressure and MAFLD was partially mediated by smoking and drinking.

Currently, there is no effective treatment for MAFLD. Our study showed that psychological factors are tightly associated with MAFLD, which suggests that we should pay attention to the mental health of patients while treating MAFLD itself. On the one hand, early screening of mental disorders, including perceived stress, and early psychological intervention may be beneficial for the early prevention of MAFLD. On the other hand, appropriate psychological intervention while treating patients who have been diagnosed with MAFLD may improve the therapeutic efficacy of treatment for MAFLD. Therefore, we believe that it is necessary in clinical practice to actively evaluate the mental health status of patients in groups at high risk of MAFLD for early prevention and intervention.

Although there are several key findings in our study, there are also several inherent limitations. First, only ultrasound was used to evaluate MAFLD in this study. Due to the limitations of ultrasound examination and individual differences between populations, there is a possibility of inaccurate diagnosis in patients with low liver fat content. However, in clinical practice, ultrasound screening of asymptomatic individuals is often used to diagnose MAFLD. CAP is the easiest, cheapest option for a quantitative assessment. We acknowledged that the lack of quantitative steatosis measurement (e.g., CAP) reduces diagnostic precision and may underestimate the relationship between perceived stress and disease severity. The study did not provide information on the association between quantitative steatosis measurement and stress, which may in turn lead to a long-term risk of MAFLD and may also change the reported association between stress and MAFLD. Second, smoking, physical activity, and diet data were obtained through standardized questionnaires, which can lead to recall bias. Third, while our mediation analysis provides insights into potential pathways, residual confounding (e.g., genetic predisposition, dietary habits) may still influence the results. Although sensitivity analyses suggested robustness to moderate unmeasured confounding, future longitudinal studies with repeated measures of stress, behaviors, and MAFLD progression are needed to strengthen causal inference. Finally, while we adjusted for key covariates (e.g., BMI, metabolic markers), unmeasured factors such as dietary patterns (e.g., high sugar/fat intake), sleep disorders, or untreated depression/anxiety—which are strongly linked to both stress and MAFLD—could residually confound our results. For instance, depression is associated with perceived stress (OR ~ 2–3) and MAFLD (OR ~ 1.5–2.0), potentially inflating our effect estimates. The *E*-value analysis suggests that such confounders could plausibly alter our conclusions, highlighting the need for future studies with comprehensive adjustment for psychosocial and lifestyle factors. Future studies should consider the importance of incorporating lifestyle risk factors and data on common fatty liver diseases to determine reliable and robust risk estimates in this population.

In conclusion, we found that perceived stress was potential associated with MAFLD. The mediation analysis revealed that the association between perceived stress and MAFLD was partially mediated by smoking and drinking. We next need to conduct longitudinal cohort studies to further clarify the causal mediating role of smoking and drinking between perceived stress and MAFLD. This study implies the necessity of actively evaluating the mental health status of patients at high risk of MAFLD and incorporating psychological approaches in the treatment of MAFLD. Our findings will provide an important reference for the early prevention and clinical treatment of MAFLD in the future.

## Data Availability

The raw data supporting the conclusions of this article will be made available by the authors, without undue reservation.

## Supplementary material

Supplementary Figure S1 Flow chart of the study population.

Supplementary Table 1 Analysis to evaluate the combined effect of smoking and drinking.
