# Supplementary figures and images for: Association between perceived stress and MAFLD partially mediated by smoking and drinking
Source: Front Med (Lausanne). 2025 Jul 29;12:1569992. doi: 10.3389/fmed.2025.1569992 (PMC12339455; doi:10.3389/fmed.2025.1569992)

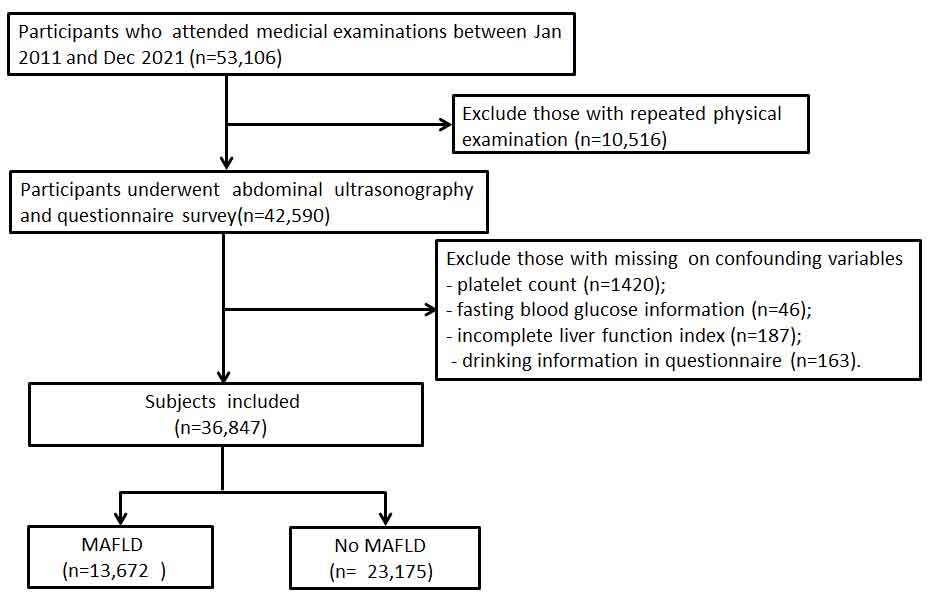

Supplement: Supplementary Figure S1 — Flow chart of the study population. [file Image_1.jpeg]
