# Supplementary material for: Association between perceived stress and MAFLD partially mediated by smoking and drinking
Source: Front Med (Lausanne). 2025 Jul 29;12:1569992. doi: 10.3389/fmed.2025.1569992 (PMC12339455; doi:10.3389/fmed.2025.1569992)
Supplement: Supplementary Table 1 — Analysis to evaluate the combined effect of smoking and drinking. [file Table_1.docx]

**Supplement table1. Analysis to evaluate the combined effect of smoking and drinking**

| Behavior | OR (95% CI) | *P*-value |
| --- | --- | --- |
| None | 1 |  |
| Smoking | 1.108（0.913-1.135） | 0.745 |
| Drinking | 1.217（1.137-1.302） | <0.001 |
| Smoking + Drinking | 1.341（1.243-1.448） | <0.001 |
